# Vector-control personnel’s knowledge, perceptions and practices towards insecticides used for indoor residual spraying in Limpopo Province, South Africa

**DOI:** 10.1186/1756-3305-6-118

**Published:** 2013-04-23

**Authors:** Khumbulani W Hlongwana, Edison J Mavundza, Elda P Mohapi, Phillip Kruger, Jasson Urbach, Samson Mukaratirwa, Rajendra Maharaj

**Affiliations:** 1 Malaria Research Unit, Medical Research Council, 491 Ridge Road, Overport, Durban, 4001, South Africa; 2 Malaria Control Programme, Department of Health, P.O. Box 33, Tzaneen, Limpopo, 0850, South Africa; 3 Africa Fighting Malaria, P.O. Box 17156, Durban, Congella, 4013, South Africa; 4 Biological and Conservation Sciences, University of KwaZulu-Natal, Private Bag X54001, Durban, 4000, South Africa

**Keywords:** Dichlorodiphenyltrichloroethane (DDT), Indoor residual spraying (IRS), Insecticides, Malaria, Vector-control workers, Knowledge, Attitudes and practices (KAP)

## Abstract

**Background:**

Contradictory arguments regarding the benefits and harm of insecticides, especially DDT, have caused concerns in different societal circles, threatening to undermine the achievements of the indoor residual spraying (IRS) programme in South Africa. These concerns were exacerbated by the screening of a documentary on South African Broadcasting Corporation (SABC) Television with anti-DDT sentiments. Consequently, Limpopo Malaria Control Programme (LMCP) Management advocated for an investigation to determine the potential effect of such campaigns on vector-control personnel’s knowledge and perceived effects of insecticides on human health, with a view to improving the educational materials designed for use in training vector-control personnel.

**Methods:**

The study was a cross-sectional descriptive survey using a structured field-piloted questionnaire, administered to 233 randomly selected vector-control personnel. Ethical clearance was granted by the University of KwaZulu-Natal. Approval for the study was granted by the Department of Health, Limpopo. Participation in the study was voluntary and all respondents signed informed consent. Descriptive statistics were used to analyse the collected data.

**Results:**

Most respondents (96.6%) had a positive perception of IRS as a method to control malaria. Despite their positive perception, 93.6% viewed IRS insecticides to be potentially harmful to the users. DDT was perceived to cause long-term reproductive and respiratory effects, whereas alpha-cypermethrin and deltamethrin were largely associated with skin irritation/itchiness and skin burn. Study participants were more worried about DDT’s potential effects on their reproductive system, including poor sexual performance, decline in libido, miscarriage and bearing children with genetic defects. However, none reported personal experience of bearing a child with genetic defects or miscarriage.

Most anti-insecticide messages, especially relating to DDT, emanated from sources external to the LMCP, mainly through radio (62%) and television (33.9%) and about 70% believed such messages. While most respondents preferred to work with a moderately itchy deltamethrin, DDT was admittedly the most effective insecticide.

**Conclusion:**

Vector-control personnel faced health and ethical dilemmas, in that, while they perceived insecticides used for IRS in Limpopo to be potentially harmful to the health of users, as purported through media, they also viewed IRS using insecticides to be effective in controlling malaria.

## Background

Over the years, concerted efforts to fight malaria have yielded positive results. Nonetheless, malaria remains a major cause of morbidity and mortality in tropical and subtropical regions of the world, accounting for approximately 225 million cases and 781,000 deaths, globally [1], 78% of which occur in sub-Saharan Africa [2]. The most vulnerable groups are children under 5 years and pregnant women [3]. In South Africa, malaria transmission is currently confined to the low-altitude areas of Limpopo, Mpumalanga and KwaZulu-Natal provinces, along the borders with Mozambique, Zimbabwe and Swaziland, whereas North-West province experiences sporadic transmission along the Molopo River [4,5]. Transmission is distinctly seasonal and limited to the warm and rainy months [5] with *Plasmodium falciparum* being the most prevalent parasite, accounting for approximately 95% of all malaria cases [4].

Achievements in malaria reduction have, mainly, been through numerous vector-control interventions, including indoor residual spraying (IRS) [6,7]. IRS is defined as the application of insecticides on the walls and ceilings of residential structures in malarious areas in order to kill and/or repel the adult vector mosquitoes that land and rest on these surfaces [7,8]. The World Health Organisation (WHO) recommends the use of four insecticide groups, namely, organochlorides, organophosphates, carbamates and pyrethroids, for IRS [8]. In South Africa, Malaria Control Programmes (MCPs) use insecticides from pyrethroid and organochlorine groups, due to their cost-effectiveness [9] and durability of residual efficacy [8]. Dichlorodiphenyltrichloroethane (DDT) is a commonly used organochloride across all malaria endemic provinces. However, the types of pyrethroids used differ from province to province. This study was conducted at a time when Limpopo was transitioning from the use of alpha-cypermethrin to deltamethrin.

Since its introduction into public health and malaria control in 1946, DDT has been effective in reducing malaria morbidity and mortality in South Africa [10] and was also lauded for its contribution in eradicating malaria in the United States, Japan, Korea, Taiwan, Spain, Italy, the Balkans, Greece and Northern Africa during the Global Malaria Eradication Programme (GMEP) of 1955-1969 [11,12]. More than 50 years later, DDT is still considered effective in reducing malaria. Despite its historical and more contemporary success stories, arguments that DDT has harmful effects on the environment and human health are unabated. Other scholars contend that such arguments fail to satisfy the basic epidemiologic criteria to prove that DDT can cause harm to human health [9].

Controversies around the use of DDT for IRS in malaria control led to a sustained pressure for its discontinuation [9,13]. Ultimately, DDT was discontinued and replaced with pyrethroids during the periods 1996 – 2000 in KwaZulu-Natal. In Limpopo, the use of DDT was also markedly reduced during the period 1998 – 2000, however, the use was not completely discontinued. Consequently, the average monthly malaria cases in KwaZulu-Natal increased considerably from 600 to over 2000 cases [14,15], resulting in DDT being re-introduced in 2000. While the return of DDT use to control malaria rekindled victory prospects against malaria, this move did not vindicate DDT from the earlier concerns that it had devastating impact on wildlife and human health.

Contentions against DDT, among other things, were the assertion that it had a widespread and devastating impact on wildlife and human health [16]. In her ground-breaking book, Rachel Carson [17] initially argued that DDT led to reproductive failures and later on, DDT was linked to weakening the eggshells of the American eagle (*Haliaetus leucocephalus*), arguably contributing to a sharp decline in this species [16]. Subsequently, a number of studies were conducted to investigate the effects of DDT on, among other things, breast milk [18], male fertility [19] and liver functions [20]. However, DDT has a notably low acute toxicity, but because of its chemical stability, it accumulates in the environment through food chains and in tissues of exposed organisms, including people living in insecticide treated structures. This has given rise to concerns relating to possible long-term toxicity to human and wildlife [8]. While a wide range of reproductive effects were reported in laboratory animals, epidemiological data did not support these findings in humans [8,21].

In contrast to DDT, the use of pyrethroids in malaria control is not fiercely contested in South Africa and elsewhere in the world, since all pyrethroids are considered to be biodegradable and are of low mammalian toxicity [22]. Despite limited attention paid to pyrethroids, these insecticides still present other problems of public health interest in the context of a continual fight against malaria. These problems include pyrethroid resistance in *An*. *funestus*[13] as discovered in KwaZulu-Natal [14] and in *An*. *gambiae s*.*s*. in West and Central Africa [23] and subsequently in Uganda [22]. Pyrethroids are overused, as they are still prominent pesticides in the agricultural sector and are also used to control malaria through IRS [24]. No suitable alternative has been found yet for impregnating bednets, thus imposing a severe strain on the insecticides [10,14].

The benefits and harm of using insecticides, and DDT in particular, did not only unfold at academic platforms, but they were taken to various media formats targeting a broader audience, especially in malarious areas. For example, a documentary with anti-DDT sentiments was screened on South African Broadcasting Corporation (SABC) Television Channel 3 on the 27^th^ of July 2010, with key interviewees residing in Limpopo. Villagers in malarious areas of Limpopo, including vector-control personnel, were also reached through road shows warning about the potential negative health effects of DDT on human health. While there is a plethora of contradictory evidence inundating various media and other communication formats around insecticides used for IRS, there is no suggestion that studies on vector-control personnel’s knowledge, perceptions and practices towards these insecticides have been conducted in South Africa.

In the light of these polarised and often ethically charged widely publicised debates around IRS insecticides, LMCP managers advocated for an investigation to determine vector-control personnel’s knowledge, practices and perceived effects of DDT and other insecticides on human health, with a view to improving the educational materials designed for use in training vector-control personnel.

## Methods

The study was conducted as a cross-sectional descriptive survey using a structured field-piloted 39 point questionnaire. The questionnaire was administered by three experienced scientists to 233 randomly selected vector-control personnel involved in IRS activities. Assuming that 60% of the 323 permanent vector-control personnel in Limpopo had received anti-IRS insecticide message(s) outside the LMCP within two years preceding the survey, with an error margin of 3.5% at a Confidence Level of 95%, we calculated that 227 participants were required for this study. Open-Epi was used to calculate the sample size. Participation in the study was voluntary and all respondents signed informed consent, which was in three vernacular languages used in Limpopo (SePedi, XiTsonga and TshiVhenda). Collected data were double entered into access database and analysed through Minitab 16 statistical software. Descriptive statistical analysis was carried out, focussing on the research participants’ knowledge and perceptions of insecticides, and their perceived potential effects on human health, including their understanding and use of protective equipment. One proportion test was performed using a statistical significance of 95% Confidence Intervals. The study obtained ethical clearance from the University of KwaZulu-Natal Humanities Faculty Ethics Committee. Subsequently, administrative approval was granted by the Limpopo Provincial Department of Health.

## Results

### Biographical information

The age of the respondents ranged from 24 to 65 years with a median age of 54 years (SD = 8.7), and a majority were over 55 years (Table 1). More specifically, 18.5% were 60 years or older and had vast vector-control experience in the LMCP. Respondents’ experiences ranged from 1 to 39 years, with the mean experience of 22.3 years (SD = 9.5). About 33.9% of the respondents had worked in the programme for 11 to 20 years and few (12.4%) had worked for 10 years or less. Notably, more than half of the respondents had over 20 years of experience in the LMCP. The majority of vector-control personnel (94.8%) worked in their home districts. Malaria control remains a male (96.6%) dominated activity and spray operators constituted the majority (68.9%) of respondents. About half of the respondents had primary level education (Table 1).

**Table 1:** Respondents’ biographical information (n = 233)

**Characteristics**	**No of respondents**	**Percentage (%)**
**Age in years**		
<36	12	5.2
36-45	43	18.5
46-55	85	36.5
>55	93	39.9
**Gender**		
Male	225	96.6
Female	8	3.4
**Highest level of education attained**		
No formal education	3	1.3
Primary	116	49.8
Secondary	95	40.8
Post-matric	18	7.7
Night school/Abet	1	0.4
**Job designation**		
Spray operator	159	68.9
Environmental health officer	4	1.7
Foreman	37	15.9
Team leader	23	9.9
Case investigator	8	3.3
Other	2	0.9
**Years of experience**		
01 – 10	29	12.4
11 – 20	79	33.9
21 – 30	66	28.3
31 – 39	57	24.4
Forgotten	2	0.9
**Work district**		
Mopani	125	53.6
Vhembe	108	46.4
**Home district**		
Capricorn	5	2.2
Mopani	121	51.9
Vhembe	105	45.1
Other	2	0.9

### Knowledge and perception of IRS and insecticides

All respondents knew the commercial names of insecticides used for IRS in Limpopo, namely, DDT and K-Othrine (deltamethrin), which substituted Fendona (Alpha-cypermethrin). Most respondents had a positive perception of IRS as a method to control malaria and neither levels of education (Table 2) nor job designation (Table 3) had clear influence on the vector-control personnel’s perception of IRS using insecticides. Very few respondents (2.1%) viewed IRS using insecticides as a bad method to control malaria. Despite the overall positive perception of vector-control personnel towards IRS using insecticides, over 93% perceived these insecticides to be potentially harmful to the users, whilst only 5% felt that it was safe to use these insecticides. Alpha-cypermethrin (72.5%) was listed as the most harmful insecticide, followed by DDT (57.1%) and deltamethrin (34.8%). The respondents’ perception of harmfulness was mainly based on their personal observation/experience (87.1%) (Table 4), in which they cited skin reaction upon contact with insecticides (alpha-cypermethrin and deltamethrin). Despite the perception that DDT caused long-term reproductive and respiratory effects, 93.6% of the respondents were of the view that DDT is effective in preventing malaria.

**Table 2:** Respondents’ perception of IRS with insecticides as a malaria control method stratified with levels of education

**Highest level of education attained**	**Perception of IRS with insecticides as malaria control method**				
	**Excellent**	**Good**	**Undecided**	**Bad**	**Total**
No education	3	0	0	0	3
Row%	100.0	0.0	0.0	0.0	100.0
Column%	1.6	0.0	0.0	0.0	1.3
Primary School education	100	12	1	3	116
Row%	86.2	10.3	0.9	2.6	100.0
Column%	52.1	36.4	33.3	60.0	49.8
Secondary School education	79	13	1	2	95
Row%	83.2	13.7	1.1	2.1	100.0
Column%	41.1	39.4	33.3	40.0	40.8
Post-matric qualification	10	7	1	0	18
Row%	55.6	38.9	5.6	0.0	100.0
Column%	5.2	21.2	33.3	0.0	7.7
Night school/Abet	0	1	0	0	1
Row%	0.0	100.0	0.0	0.0	100.0
Column%	0.0	3.0	0.0	0.0	0.4
**Total**	192	33	3	5	233
**Row%**	82.4	14.2	1.3	2.1	100.0
**Column%**	100.0	100.0	100.0	100.0	100.0

**Table 3:** Respondents’ perception of IRS with insecticides as a malaria control method stratified with job designation

**Job designation**	**Perception of IRS with insecticides as malaria control method**				
	**Excellent**	**Good**	**Undecided**	**Bad**	**Total**
Spray Operator	133	20	3	3	159
Row%	83.6	12.6	1.9	1.9	100.0
Column%	69.3	60.6	100.0	60.0	68.2
Environmental Health Officer	4	0	0	0	4
Row%	100.0	0.0	0.0	0.0	100.0
Column%	2.1	0.0	0.0	0.0	1.7
**Foreman**	28	8	0	1	37
Row%	75.7	21.6	0.0	2.7	100.0
Column%	14.6	24.2	0.0	20.0	15.9
Team Leader	19	4	0	0	23
Row%	82.6	17.4	0.0	0.0	100.0
Column%	9.9	12.1	0.0	0.0	9.9
Case investigator	6	1	0	1	8
Row%	75.0	12.5	0.0	12.5	100.0
Column%	3.1	3.0	0.0	20.0	3.4
Other	2	0	0	0	2
Row%	100.0	0.0	0.0	0.0	100.0
Column%	1.0	0.0	0.0	0.0	0.9
**Total**	192	33	3	5	233
**Row%**	82.4	14.2	1.3	2.1	100.0
**Column%**	100.0	100.0	100.0	100.0	100.0

**Table 4:** Sources for the respondents’ perceived harmfulness of insecticides used in Limpopo (n = 233)

**Sources**	**X**	**Sample p**	**95% CI**
Personal observations/experience	203	87.12	82.13 – 91.14
Information from friend/colleague	33	14.16	9.95 – 19.31
Householders/community members	11	4.72	2.38 – 8.29
Scientific reports/publications	3	1.29	0.27 – 3.72
Media reports	5	2.15	0.70 – 4.94
Other	1	0. 43	0.01 – 2.37

Despite the respondents’ perceived harmfulness and effectiveness of insecticides used in Limpopo, 94% disliked spraying with certain insecticides, including alpha-cypermethrin (73.8%), DDT (51.1%) and deltamethrin (30%) (Table 5). Of the 51.1% of respondents who did not like some aspects about DDT, the reasons included the perceptions that it causes respiratory illnesses (37.8%), reproductive health conditions (26.1%) and that the communities where IRS operations are conducted, dislike it (14.3%). Of the 73.8% of respondents who did not like some aspects of alpha-cypermethrin, their concerns were mainly skin irritation/itchiness (79.7%) and skin burn (25%). Deltamethrin was also linked to moderate skin irritation/itchiness (70%), moderate skin burn (22.9%) and respiratory illnesses (12.9%).

**Table 5:** Insecticides disliked by vector-control personnel (multiple options allowed) (n = 233)

**Insecticides**	**X**	**Sample p**	**95% CI**
DDT	119	51.07	44.46 – 57.66
Alpha-cypermethrin	172	73.82	67.68 – 79.34
Deltamethrin	70	30.04	24.23 – 36.37

### Messages and messaging of insecticide related information or knowledge

A significantly greater proportion (73.4%) of respondents received messages about insecticides outside LMCP within two years preceding the survey, compared with those who did not receive messages (26.6%). The observed proportion who received messages was significantly different from the hypothesized proportion of 60% (P < 0.0001; 95% CI: 67.2% - 78.9%). Eighty percent of the time, messages were anti-insecticides, especially anti-DDT (96.5%). The main sources of message dissemination were radio (62%) and television (33.9%). About 70% of those who received messages believed them, whereas 16% and 14% partially believed and did not believe the messages, respectively. Despite the messaging on insecticides and the respondents’ perception of insecticides, including harmfulness and effectiveness, a majority preferred working with deltamethrin (75.1%), due to its moderate skin itchiness/irritation, moderate skin burn, and the positive feedback from the community.

**Figure 1:**
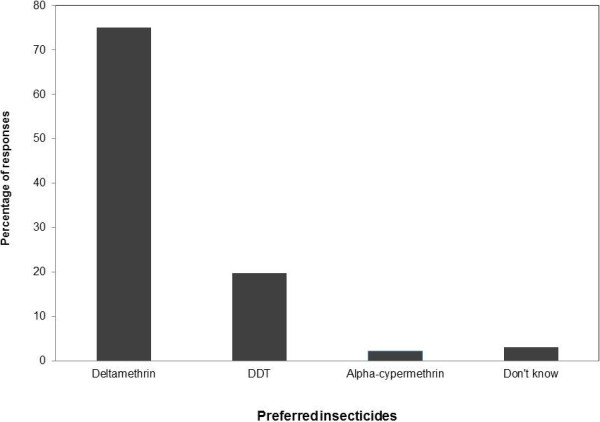
Respondents’ preferred insecticides for the indoor residual spraying.

DDT and alpha-cypermethrin were preferred by 19.7% and 2.2%, respectively (Figure 1). Information shared by vector-control personnel was that they were in agreement with householders on the intensity of skin itchiness/irritation associated with the use of alpha-cypermethrin. In support of this assertion, respondents cited community reports whereby their children complained about itchy skin immediately after leaning against the house walls sprayed with alpha-cypermethrin.

Most respondents (83.3%) knew nothing about IRS insecticides and were not worried about working with them when they joined LMCP. However, with time, there was a noteworthy shift in the respondents’ perception of working with insecticides. Vector-control personnel became increasingly worried about working with insecticides and according to their retrospective memory the shift was more than five-fold at the time of a survey compared to when they first joined the LMCP. At the time of a survey, at least, 30.9% were still not worried about working with insecticides and 75% of them were older than 45 years.

Prior to joining the LMCP, 99.1% of the respondents either described their health as very healthy or just healthy. However, at the time of a survey, about 62.2% described themselves as either unhealthy or very unhealthy, and about 91% attributed the reportedly ill state-of-health to their physical contact with insecticides. Respondents drew no connection between their ill state-of-health and age. Respiratory illnesses, poor eyesight, reproductive conditions, body pains and fatigue featured prominently as the key health problems they experienced.

### Knowledge and attitudes towards personal protective equipment and insecticides

About 94% of the respondents thought that personal protective equipment (PPEs) were necessary when conducting IRS. Only 2.6% viewed PPEs as more of an impediment, stating that insecticides spread over visors and obscure the view while conducting spraying. At least, all respondents in favour of PPEs cited skin protection as the key benefit. However, some concerns were raised regarding the quality of PPEs, with a specific reference to masks, visors, caps and shields.

### Vector-control personnel’s overall impression of IRS in Limpopo

Through open-ended questions, respondents were asked to list all health risks they associated with physical contact with insecticides. The perceived health risks included: respiratory illnesses, headache, skin diseases, diarrhoea, reproductive health, poor eyesight and fatigue. Respiratory illnesses included tuberculosis, asthma and pneumonia, whereas reproductive conditions were mainly poor sexual performance, declining libido, women giving birth to children with genetic defects and miscarriage. However, despite all the risks listed by respondents, none reported personal experience of giving birth to a child with genetic defects or miscarriage. Few respondents believed that insecticides penetrate the operator’s skin and poison the blood.

Respondents were also asked to identify areas in IRS which, in their opinion, needed some improvements. Few themes emerged from the suggested improvements, namely, compensation, choice of insecticides, information, education and communication (IEC), living and working conditions, health and hygiene, as well as staff recruitment and supervision. With regards to compensation, it was noteworthy that almost all respondents consistently mentioned what they referred to as ‘danger allowance’ arguing that they work with dangerous substances, hence they pleaded for the risk exposure compensation. In defence of their call for ‘danger allowance’, respondents further mentioned that DDT is labelled a ‘slow poison’ on its cover.

While most respondents could not refute DDT’s long lasting residual efficacy, they argued that IRS would benefit from a scientific discovery of an alternative insecticide to replace DDT. Others felt that they needed more education on toxicological profiles of insecticides. Vector-control personnel’s experience of IRS refusal rate was also seen as an indication of the need to enhance IEC to counter anti-insecticide messages in malarious communities.

Respondents also proposed that malaria camps, store rooms and equipment be better maintained. Most respondents argued for extra pairs of PPEs. Few respondents suggested adjustments in spray hours to enable them to start early in the morning and finish before temperatures rise high. There was also a suggestion to employ safety officers into the vector-control workforce. Almost all respondents proposed that all staff working with insecticides be subjected to regular medical check-ups to establish whether or not insecticides had effects on their health. There was a proposal that spray operators drink milk shortly after spraying to minimise health risks. Respondents suggested that the recruitment of temporary spray operators be subjected to the normal recruitment processes and not the politically appointed local councillors as per the current practice.

## Discussion

Vector-control personnel face health and ethical dilemmas, in that, while they perceive insecticides used for IRS in Limpopo to be potentially harmful to their health, they also believe that IRS using insecticides, especially DDT, is effective in controlling malaria. In their experience in IRS, which ranged between 1 and 39 years, there were fewer reported malaria cases when they sprayed with DDT. Effectiveness of DDT has also been widely confirmed by the literature [10,11]. This study has revealed that householders and vector-control personnel are in agreement on the effectiveness of DDT, despite all the other insecticide-related concerns and controversies in the public domain.

The respondents’ perception of harmfulness was based on two critical factors, namely, perceived bodily reaction upon contact with insecticide and perceived long-term health effects. Perceived bodily reaction upon contact with insecticide was consistently linked to pyrethroids, namely, alpha-cypermethrin and deltamethrin, whereas perceived long-term health effects were associated with DDT. Several outbreaks of acute intoxication due to spray operators’ dermal exposure to pyrethroid compounds was reported in China in 1982, pointing to the fact that the main route of pyrethroid absorption is through the skin [25]. The results of this study are congruent with the literature regarding absorption of insecticides through physical chemical contact with the skin, qualifying the need for training of spray operators on appropriate, proper and consistent use of PPEs, in line with the safety instruction regulations. Spray operators’ safety precautions also depend largely on personal hygiene, including washing PPEs regularly, as per IRS guidelines. Confined spaces also play a critical role in increasing inhalation of pyrethroids [26], which justifies the need for consistent use of masks when applying insecticides.

On the other hand, the respondents’ views on DDT’s harmfulness were largely based on the information they received through the media. The study conducted by Yates and Stroup [27] on media coverage and EPA pesticide decisions concluded that media coverage does influence public opinion on whether or not to support a pesticide in question. For example, it was a dramatisation of potential danger from Alar pesticide that swayed public opinion in favour of eliminating its use [27]. In this study, the media had a huge impact on how vector-control personnel perceived insecticides and DDT in particular. For example, most respondents who had received messages from the media described the content of the messages as ‘anti-insecticide’. This focuses on two issues, namely, the media as a powerful tool in shaping public opinions, and the publicity biased views of the media on DDT. This bias was evident when malaria operational research experts and malaria control personnel did not participate in the screening of a documentary with anti-DDT sentiments.

While DDT is known to have a low acute toxicity, because of its chemical stability, it accumulates in the environment through food chains and in tissues of exposed organisms; including people living in insecticide treated structures [28]. This has given rise to concern regarding possible long-term toxicity in humans [8]. Whereas a wide range of effects were reported in laboratory animals, epidemiological data did not support these findings in humans [8,21]. Subsequently, questions about the long-term harmfulness of DDT were raised by the media after the publication of a study on urogenital birth defects conducted in Vhembe, Limpopo Province [29], where DDT remains the mainstay of IRS. Most importantly, none of the study respondents reported to have been diagnosed with any of the alleged health conditions. The only condition they could link with the effects of long-term exposure to DDT was their alleged deteriorating sexual performance. This was despite their average age being about 52 years. The study conducted by Polsky and colleagues [21] found no relationship between plasma levels of organochlorines and erectile dysfunction risks amongst humans, despite earlier associations established through experiments done on rats.

Evidence from the literature suggests that older men are at greater risk of experiencing sexual dysfunctions, including erectile dysfunction [30,31], reduced libido [32] and reduced sexual satisfaction [33]. In addition, old age is strongly associated with signs and symptoms, such as muscle weakness and fatigue [34]. In a cohort study conducted in the United States [30], there was a sharp increase in the percentage of men who experienced their first problems with erection after 50 years of age. Despite the respondents’ median age being 54 years in this study, vector-control personnel solely attributed their sexual problems to insecticides. Ageing is also generally associated with decreased muscle strength, fitness and loss of functional capacity [32]. Consistent with the findings of other studies, this study revealed that, irrespective of age, men hardly stop worrying about their experiences of erectile dysfunction [35].

Similar to other studies [36], this study also found that vector-control personnel were less concerned about deltamethrin because of moderate harmfulness in comparison to alpha-cypermethrin. Congruent with the findings of this study, Luty *et al*. [36] noted that human dermal exposure to alpha-cypermethrin occurs during activities such as preparation of the working solution, spraying and washing of IRS equipment. Typical symptoms of acute exposure to alpha-cypermethrin are irritation of skin and eyes, headaches, dizziness, nausea, vomiting, diarrhoea, excessive salivation and fatigue [36]. At least, findings relating to alpha-cypermethrin are consistent with what has been found by other studies. Study respondents thought that alpha-cypermethrin was the most harmful insecticide, based on the severity of symptoms of acute exposure. Most respondents were in agreement that PPEs are essential to protect them against direct exposure to insecticides.

Despite increasing and influential anti-DDT messages, respondents were more concerned about the short-term impact of a barely publicised yet highly disfavoured alpha-cypermethrin. In addition to their personal experiences and views, respondents’ preference of insecticides was also based on whether or not the community liked it. Anti-DDT messages gained trustworthiness for two fundamental reasons: (1) the source of information was scientists and (2) most popular and trusted media (radio and television) were used. This may actually be indicative of worthwhile options for LMCP to have impactful IEC. It may also be necessary to draw from The Health Belief Model (HBM) and the Stages of Change (Transtheoretical) Model widely used in social psychology to describe messaging and behaviour change [37,38] when developing and implementing IEC messages [37]. This shows that communication and messaging are complex processes influenced by factors such as professional standing and credibility of the communicator and the communication channels used.

The respondents’ suggestions of having regular medical check-ups and compensatory allowance (danger allowance) for allegedly working with dangerous chemicals requires further investigation, especially in other settings where DDT is mainly used. The respondents’ consistent mentioning of ‘danger allowance’ in this study created an impression that there was a prior caucus prior to interviews. Some respondents would even forget this concept and recall it at a later stage of the interview process. Retirement bonus for long service in a dangerous field of work was also proposed. Conducting similar studies in other settings where IRS is implemented will give a sense of whether LMCP vector-control personnel’s expectations are widespread or peculiar to their situation. A Scientific basis for the spray operators proposal to drink milk each time they finish spraying to minimise health risks, needs to be established if such practice has to be supported. It may be useful to train health officers, spray operators, supervisors and drivers on the treatment of emergency cases of critical exposure to and poisoning by insecticides before spraying occurs. Subsequently, storage facilities and transport vehicles should be equipped with first aid kits.

## Conclusion

This study provided useful insights into the knowledge of vector-control personnel, their perception of IRS and various insecticides, including the health and ethical dilemmas they face while working with these insecticides. However, it may be beneficial for similar studies in future, to include factors such as smoking, alcohol consumption, eating habits and physical exercise of vector-control personnel when assessing their perception of health. Having a control group of people with similar characteristics, but not exposed to insecticides, may also enhance the strength of the results. Furthermore, conducting similar studies in other countries using IRS and DDT in particular may be helpful in establishing the consistency or uniqueness of vector-control personnel’s experiences in Limpopo. In summary, this study has revealed that IRS with insecticides is unlikely to yield optimal outcome, unless vector-control personnel themselves are well educated and support IRS with insecticides.

## Competing interests

The authors declare that they have no competing interests.

## Authors’ contributions

KWH, EJM and EPM were involved in all study processes including design, data acquisition, analysis and interpretation of the results as well as the drafting of the manuscript. JU, PK, RM came up with the initial study concept. Subsequently, they immensely contributed and shaped the study proposal and reviewed all drafts of the manuscripts. SM shaped the analysis and revised all drafts of the manuscript. All authors read and approved the final version of the manuscript.
